# Supplementary material for: Astrocyte-Derived Exosomes Differentially Shape T Cells’ Immune Response in MS Patients
Source: Int J Mol Sci. 2023 Apr 18;24(8):7470. doi: 10.3390/ijms24087470 (PMC10138532; doi:10.3390/ijms24087470)
Supplement: Supplementary file 1 [file ijms-24-07470-s001.zip › ijms-2320584-supplementary.pdf]

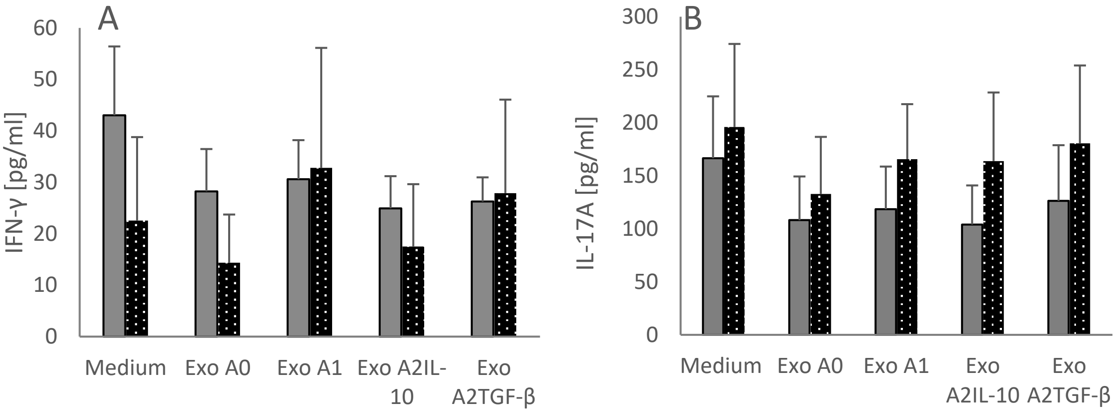

Supplement Figure S1. Comparison of IFN-γ (A) and IL-17A (B) production in CD4+ T-cell cultures from multiple sclerosis-affected patients (filled bars) and healthy donors with excluded autoimmune disease (white dotted bars). After separation from PBMC, CD4+ T-cells from every donor were isolated by magnetic sorting, and cultured on 48-well culture plates (10^6 cells/well) with exosomes isolated from non-stimulated (Exo A0), pro-inflammatory cytokines' stimulated (Exo A1) and anti-inflammatory cytokines' (IL-10, TGF-β) stimulated (respectively Exo A2IL-10, Exo A2TGF-β) human astrocytes. Data shown as mean ± SEM. Comparisons of groups were made with U Mann-Whitney rank-sum test.

A)

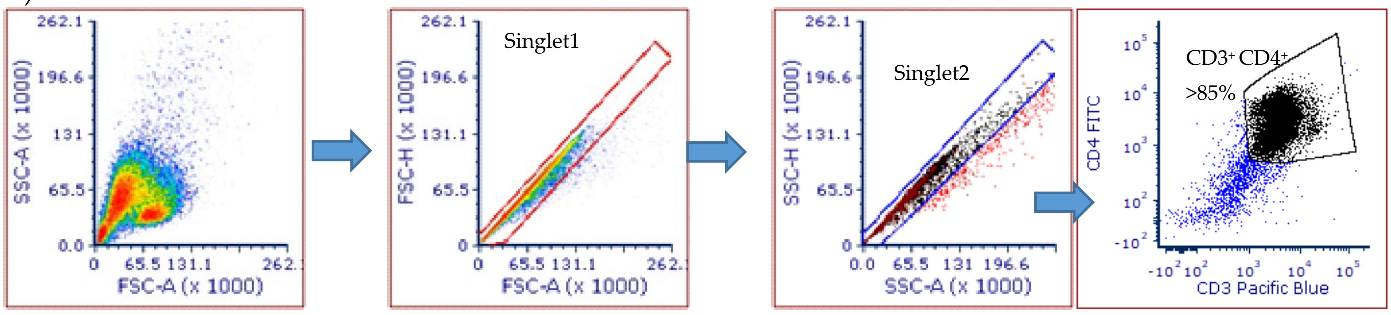
262.1
196.6
131
65.5
0.0
SSC-A (x 1000)
FSC-A (x 1000)
Singlet1
FSC-H (x 1000)
Singlet2
SSC-H (x 1000)
SSC-A (x 1000)
CD3+ CD4+
>85%
CD4 FITC
CD3 Pacific Blue

B)

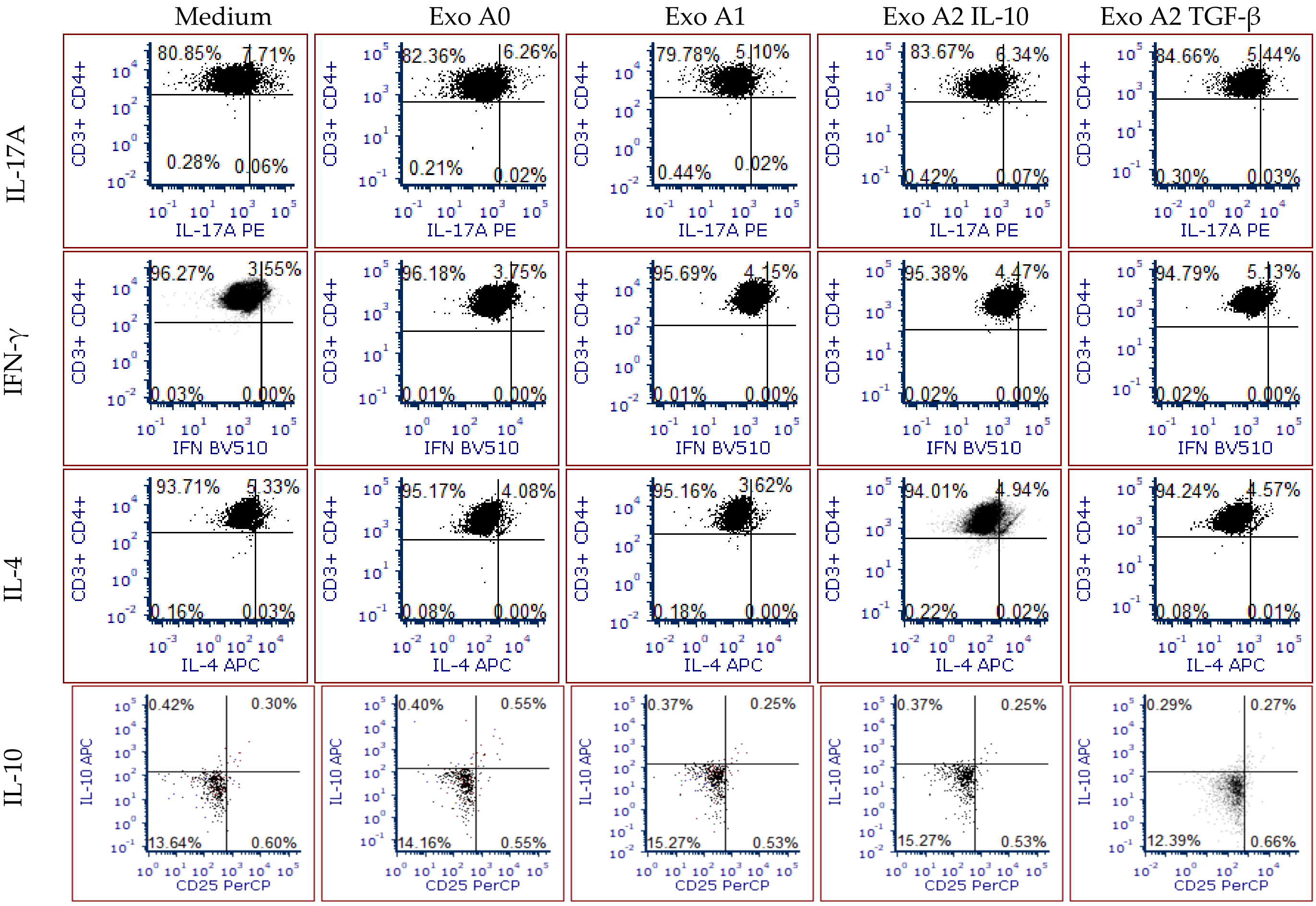
Medium
Exo A0
Exo A1
Exo A2 IL-10
Exo A2 TGF-β
IL-17A
IFN-γ
IL-4
IL-10
CD3+ CD4+
IL-17A PE
IFN BV510
IL-4 APC
IL-10 APC
CD25 PerCP
80.85% 7.71% 0.28% 0.06%
82.36% 6.26% 0.21% 0.02%
79.78% 5.10% 0.44% 0.02%
83.67% 6.34% 0.42% 0.07%
84.66% 5.44% 0.30% 0.03%
96.27% 3.55% 0.03% 0.00%
96.18% 3.75% 0.01% 0.00%
95.69% 4.15% 0.01% 0.00%
95.38% 4.47% 0.02% 0.00%
94.79% 5.13% 0.02% 0.00%
93.71% 5.33% 0.16% 0.03%
95.17% 4.08% 0.08% 0.00%
95.16% 3.62% 0.18% 0.00%
94.01% 4.94% 0.22% 0.02%
94.24% 4.57% 0.08% 0.01%
0.42% 0.30% 13.64% 0.60%
0.40% 0.55% 14.16% 0.55%
0.37% 0.25% 15.27% 0.53%
0.37% 0.25% 15.27% 0.53%
0.29% 0.27% 12.39% 0.66%

C)

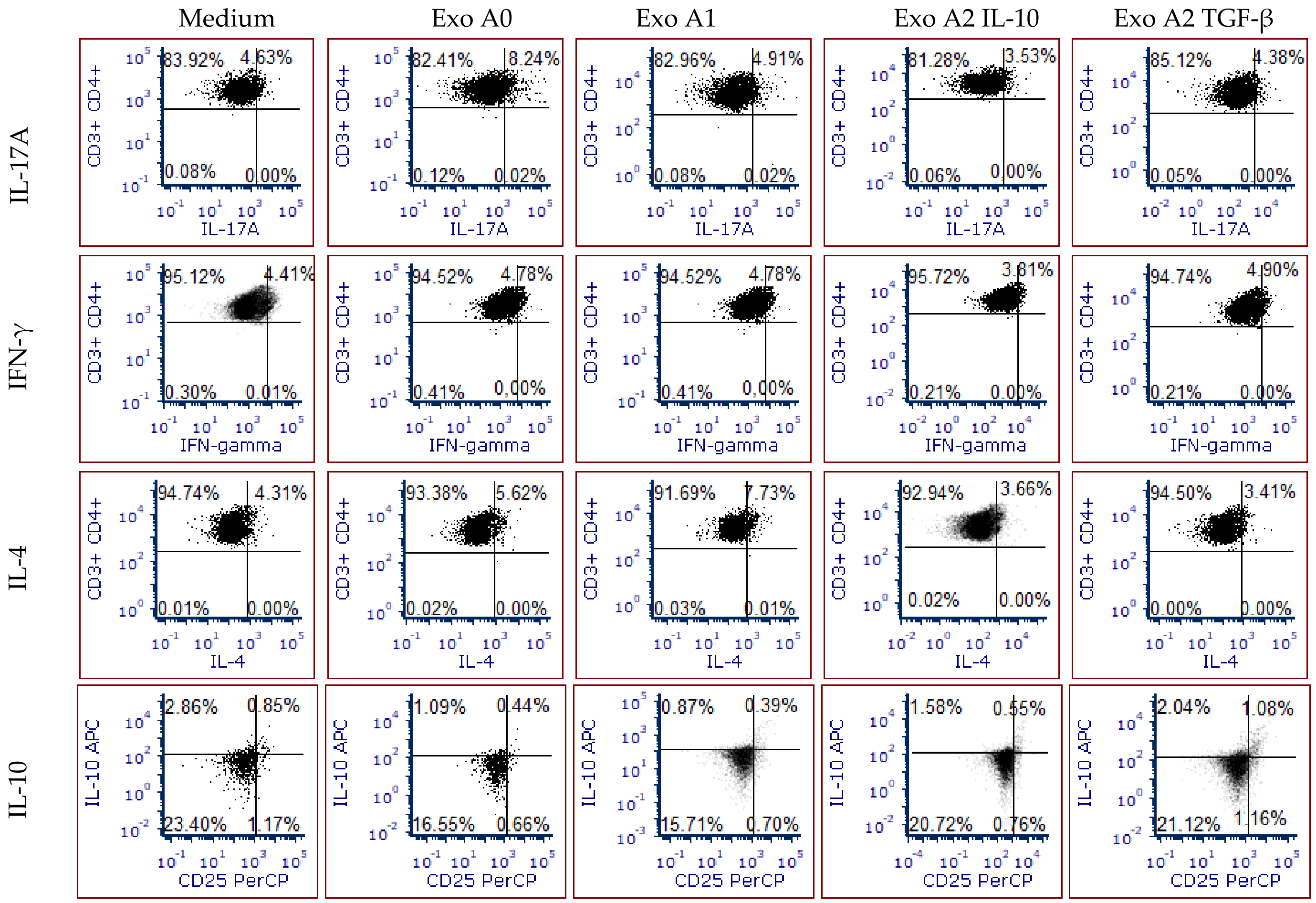

Supplement Figure S2. Representative dot plots from flow cytometry analyses of CD4+ T-cells. A) Gating strategy and elimination of cell aggregates. Percentage values of IL-17A, IFN-γ, IL-4 and IL-10 -producing T-cells from MS-affected patients (B), and non-MS donors (C), expressed as % of CD3, CD4 -positive T lymphocytes. T cells were separated from PBMC fraction after density gradient centrifugation (FicolPaquePlus), with magnetic sorting (MACS, Miltenyi Biotec) and cultured for 96h in culture medium (RPMI1640/10% FBS/penicillin, streptomycin) alone or with addition of astrocyte-derived extracellular vesicles.

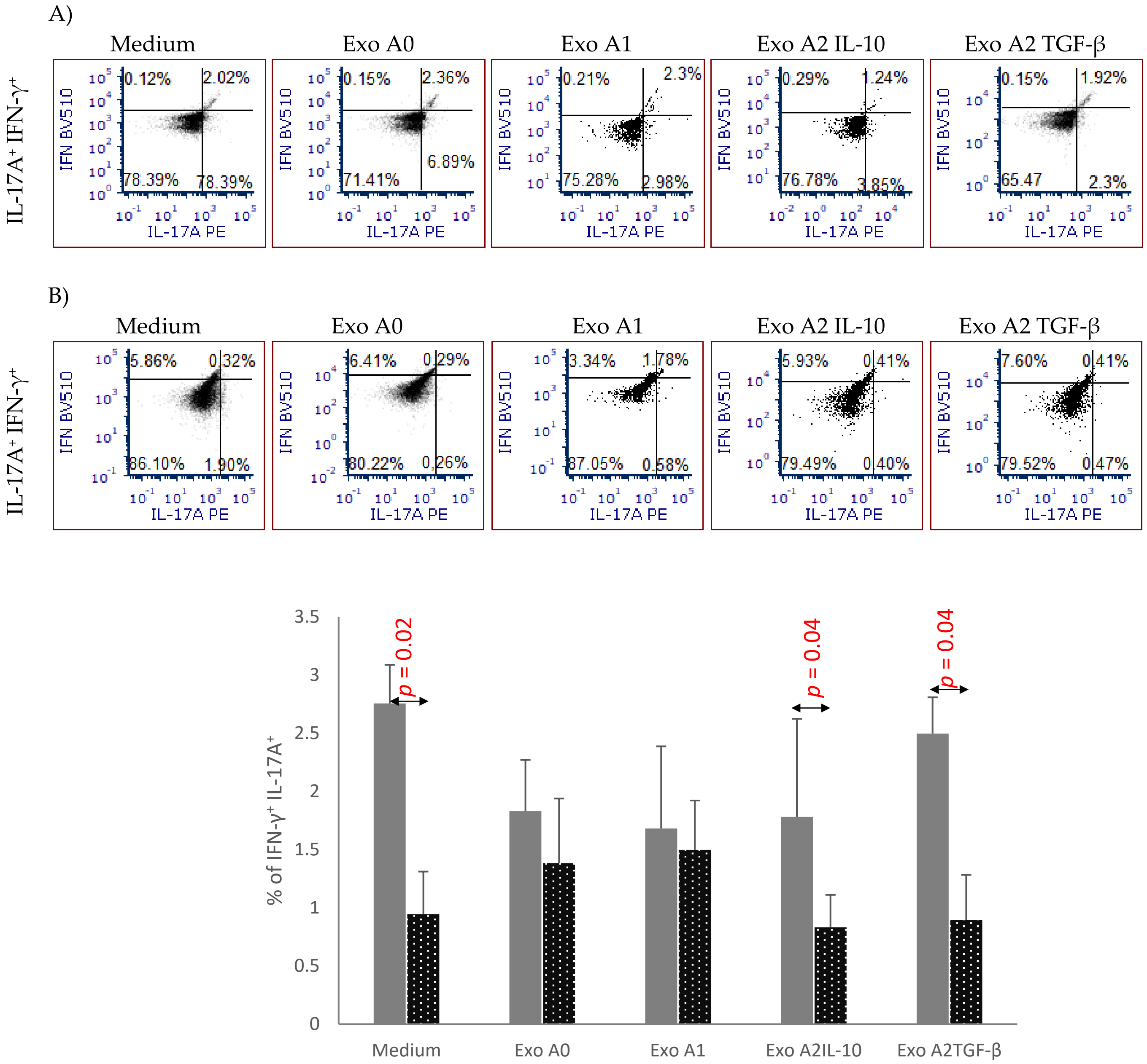

Supplement Figure S3. Results of flow cytometry analysis of double producers (IFN-γ^{+} IL-17A^{+}). Percentage values of IL-17A/IFN-γ -producing T-cells from MS-affected patients (A), and non-MS donors (B), expressed as % of CD3, CD4 -positive T lymphocytes. Mean percentage of CD3^{+} CD4^{+} cells double positive for IFN-γ and IL-17A cytokines were compared between study groups. Data for MS patients are presented with filled bars (7 persons), results for non-MS donors are in white dotted bars (4 persons). After 96h of T-cell cultures in presence of astrocyte-derived exosomes or medium alone, culture media were collected, cells were suspended in new medium containing brefeldin A for 12h, next cells were harvested and stained for flow cytometry. Cells were analysed as a % of CD3^{+} CD4^{+} cells double positive for IFN-γ and IL-17A. Data were shown as mean ± SE. Comparisons between variables were made with non-parametric Wilcoxon signed-rank test, no significant differences were observed. Comparisons between groups were made with U Mann-Whitney rank-sum test.
